# Disparities in cancer mortality patterns: A comprehensive examination of U.S. rural and urban adults, 1999–2020

**DOI:** 10.1002/cam4.6451

**Published:** 2023-08-10

**Authors:** Itunu O. Sokale, Syed Ahsan Raza, Aaron P. Thrift

**Affiliations:** ^1^ Section of Epidemiology and Population Sciences, Department of Medicine Baylor College of Medicine Houston Texas USA; ^2^ Dan L Duncan Comprehensive Cancer Center Baylor College of Medicine Houston Texas USA

**Keywords:** cancer prevention, cancer risk factors, epidemiology, gynecological oncology, liver cancer, pancreatic cancer

## Abstract

**Background:**

Cancer mortality rates overall in the U.S. have decreased significantly; however, the rate of decline has not been uniform across sociodemographic groups. We aimed to compare trends in cancer mortality rates from 1999 to 2020 between rural and urban individuals and to examine whether any rural–urban differences are uniform across racial and ethnic groups.

**Methods:**

We used U.S.‐wide data from the National Center for Health Statistics, for all cancer deaths among individuals aged 25 years or older. We estimated average annual percentage change (AAPC) in age‐standardized cancer mortality rates in the U.S. by cancer type, rural–urban status, sex, and race and ethnicity.

**Results:**

There was a larger reduction in cancer mortality rates among individuals from urban (males: AAPC, −1.96%; 95% CI, −2.03, −1.90; females: AAPC, −1.56%; 95% CI, −1.64, −1.48) than rural (males: AAPC, −1.43%; 95% CI, −1.47, −1.39; females: AAPC, −0.93; 95% CI, −1.03, −0.82) areas. AAPCs for cancer types were uniformly higher among urban areas compared with rural areas. Despite overall decreases, deaths rates for liver and pancreas cancers increased, including in the most recent period among males (2012–2020, APC, 1.34; 95% CI, 0.49, 2.20) and females (2013–2020, APC, 1.52; 95% CI, 0.03, 3.02) in rural areas.

**Conclusions:**

Cancer death rates decreased in all racial and ethnic populations; however, the rural–urban differences varied by race/ethnicity. The rate of decline in mortality rates were lower in rural areas and death rates for liver and pancreas cancers increased, particularly for individuals living in rural America.

## 1 INTRODUCTION

Cancer is a leading cause of death in the U.S.^1^ While cancer mortality rates overall have decreased significantly over recent decades,^2^ the rate of decline has not been uniform across sociodemographic groups. For example, approximately one in five Americans live in rural areas; cancer death rates are higher among rural (nonmetropolitan) populations compared to those living in urban (metropolitan) areas in the U.S.^3^ The magnitude of the differences between urban and rural populations in terms of cancer mortality rates vary by cancer site. Still, there are also important disparities in mortality rates within these two distinct populations.

Unlike non‐rural populations, rural communities have poorer outcomes, particularly for preventable cancers, due to limited access to evidence‐based interventions (including smoking cessation and cancer screening), and slower dissemination of medical advancements.^4^, ^5^, ^6^ In particular, rural areas have higher mortality rates for smoking‐related cancers (lung cancer) and cancers that can be prevented or detected earlier by screening (colorectal and cervical cancers).^3^, ^7^ Cancer screening rates are lower in rural than urban U.S.,^6^, ^8^, ^9^ and this may be exaggerated, especially among racial and ethnic minorities in rural communities. Disparities in cancer mortality remain by race/ethnicity, partially due to inequities in stage at diagnosis, and access to high‐quality therapy.^10^, ^11^ For some racial‐ethnic minorities, mainly rural subgroups, cancer death rates have declined more gradually than for non‐Hispanic whites.^7^ Studies providing comprehensive temporal overviews of age‐adjusted, race‐ and sex‐specific cancer mortality rates overall and by cancer site according to rural–urban status are scarce.^12^, ^13^, ^14^

To examine recent trends and to inform interventions aimed at addressing regional disparities in cancer death rates, we evaluated differences in secular trends in cancer mortality rates from 1999 to 2020 among rural and urban counties in the U.S. by cancer site and sex. In addition, we compared overall cancer mortality rates over time within and across rural and urban areas by race/ethnicity. These findings can be used to identify sub‐populations in the U.S. that might benefit from cancer prevention strategies and reduce cancer‐related deaths.

## 2 METHODS

### 2.1 Data source

We analyzed annual cancer mortality data from the National Center for Health Statistics (NCHS), over 21 years, from January 1999 to December 2020. Data were retrieved from the Multiple Cause of Death Files, based on data compilation from 57 vital statistics jurisdictions through the Vital Statistics Cooperative Program. Data represented in the NCHS registry includes demographic statistics (e.g., race/ethnicity, age, sex, and area of residence) and tumor characteristics (e.g., year of diagnosis, site, stage, and histology). We identified cancer‐related deaths in the file based on ICD‐10 codes C00‐C97 and cancer sites using codes shown in Table S1.

### 2.2 Statistical analysis

Our primary analysis focused on sex‐specific age‐adjusted cancer mortality rates by rural–urban classification among U.S. adults aged 25 years or older. Delineation of metro versus nonmetro cases was based on the 2013 NCHS Urban–Rural Classification Scheme for Counties,^15^ which separates counties based on their metropolitan statistical area (MSA) population. We defined metro population centers for this study as being large central metro (counties in MSAs with a population of >1 million) to small metro (counties in MSAs with a population of 50,000–250,000). Nonmetro was defined as micropolitan (MSAs with <50,000 population) to noncore counties. We also compared death rates by race and ethnicity (non‐Hispanic white [NHW], non‐Hispanic Black [NHB], Hispanic, non‐Hispanic American Indian/Alaska Native [AI/AN], and non‐Hispanic Asian or Pacific Islander [API]).

All age‐adjusted rates were standardized to the 2000 U.S. population and reported per 100,000 persons. We investigated trends in death rates over time using the National Cancer Institute's Joinpoint program.^16^ The National Cancer Institute's Joinpoint program software used for our analysis, incorporates default values (maximum of 2 joinpoints) for the number of joinpoints based on certain guidelines and recommendations.^16^, ^17^ Using default of 2 joinpoints through NCI joinpoint program helps maintain statistical robustness and prevent overfitting of the joinpoint regression model. Thus, we allowed a maximum of 2 joinpoints with a minimum of 4 observations per segment. In addition, we selected “Uncorrelated” in joinpoint software. As such, joinpoint assumes the random errors in the regression model are uncorrelated with constant variance and estimated the regression coefficients by ordinary least squares. We calculated the annual percent change (APC) for each segment and the average annual percent change (AAPC) for the study period from 1999 to 2020 using the weighted average of the slope coefficients of the underlying joinpoint regression line. Weights were equal to the length of each segment over the interval. In addition, to determine whether the APC was significantly different from zero, a *t*‐test was used. For AAPC, a *t*‐test was used for zero joinpoints, while a *z*‐test was applied for one or more joinpoints to determine if it was significantly different from zero. To examine whether the slopes of the change in trend between groups were similar (or not) in direction, we used a parallelism test. We considered trends to be statistically significant if the 2‐sided *p* < 0.05.

## 3 RESULTS

### 3.1 Cancer mortality burden

A total of 12,580,140 cancer deaths occurred between 1999 and 2020 among individuals in the U.S. aged 25 years or older. The age‐adjusted death rate was 282.7 per 100,000 population among rural persons (346.8 per 100,000 males; 235.3 per 100,000 females) and 258.2 per 100,000 population among urban persons (311.2 per 100,000 males; 221.4 per 100,000 females). Cancer site‐specific death rates are shown by rural–urban status and sex in Table 1. Specifically, the age‐adjusted death rate for lung cancer was higher among rural populations (106.1 per 100,000 males; 61.3 per 100,000 females) than in urban communities (85.4 per 100,000 males; 54.7 per 100,000 females). Similarly, rural groups had a greater burden of colorectal cancer deaths (30.6 per 100,000 males; 21.8 per 100,000 females) than their urban counterparts (26.8 per 100,000 males; 19.3 per 100,000 females), as well as cervical cancer (4.2 per 100,000 rural women; 3.6 per 100,000 urban women (Table 1).

**TABLE 1:** Age‐adjusted mortality rates for common causes of cancer death by rural–urban status and sex among persons aged ≥25 years in the United States, 1999–2020.

	Urban		Rural	
Cause of death	Males	Females	Males	Females
All malignant cancers	311.2	221.4	346.8	235.3
Female breast	N/A	34.5	N/A	34.4
Cervix uteri	N/A	3.6	N/A	4.2
Colon and rectum	26.8	19.3	30.6	21.8
Corpus and uterus, NOS	N/A	7.1	N/A	6.7
Kidney and renal pelvis	8.4	3.7	9.8	4.4
Leukemia	13.4	7.5	14.4	8.0
Liver and intrahepatic bile	13.8	5.6	12.1	5.3
Lung and bronchus	85.4	54.7	106.1	61.3
Myeloma	6.6	4.3	6.7	4.3
Non‐Hodgkin lymphoma	11.5	7.2	12.1	7.7
Ovary	N/A	11.7	N/A	11.8
Pancreas	19.4	14.8	19.5	14.6
Prostate	34.1	N/A	36.0	N/A
Stomach	7.4	4.0	6.3	3.2
Urinary bladder	11.5	3.4	11.8	3.4

*Note*: Rates are per 100,000 and age‐adjusted to the 2000 U.S. Standard Population.

Abbreviation: NOS, not otherwise specified.

### 3.2 Trends in cancer mortality

There was a more considerable reduction in cancer mortality rates among individuals from urban areas. Cancer mortality rates in urban areas decreased from 200.0 per 100,000 population in 1999 to 140.4 per 100,000 population in 2020 (absolute decrease of 59.6 per 100,000). The change in rates was greater for males (absolute decrease of 84.1 per 100,000) than females (absolute decrease of 46.2 per 100,000). In rural areas, cancer mortality rates decreased from 204.5 per 100,000 population in 1999 to 163.8 per 100,000 population in 2020 (absolute decrease of 40.7 per 100,000). As with urban areas, the change in rates was greater for males (absolute decrease of 67.2 per 100,000) than females (absolute decrease of 26.6 per 100,000).

In the joinpint analysis, between 1999 and 2020, among males, death rates for all cancers combined decreased by 1.96% (95% CI, −2.03, −1.90) per year in urban areas compared to 1.43% (95% CI, −1.47, −1.39) per year in rural areas (Table 2). For females, mortality rates for all cancers combined also decreased at a faster rate in urban populations (AAPC, −1.56%; 95% CI, −1.64, −1.48) compared with rural areas (AAPC, −0.93; 95% CI, −1.03, −0.82) (Table 3).

**TABLE 2:** Annual percentage changes in cancer death rates by rural–urban status and cancer site among **males** aged ≥25 years in the United States, 1999–2020.

Cause of Death	AAPC	Segment 1		Segment 2		Segment 3	
Urban							
All malignant cancers	**−1.96 (−2.03, −1.90)**	1999–2014	**−1.86 (−1.91, −1.81)**	2014–2020	**−2.23 (−2.43, −2.03)**	N/A	N/A
Colon and rectum	**−2.82 (−3.00, −2.64)**	1999–2011	**−3.31 (−3.54, −3.08)**	2011–2020	**−2.16 (−2.50, −1.53)**	N/A	N/A
Kidney and renal pelvis	**−1.14 (−1.49, −0.79)**	1999–2014	**−0.74 (−0.99, −0.48)**	2014–2020	**−2.15 (−3.28, −1.00)**	N/A	N/A
Leukemia	**−1.44 (−1.62, −1.27)**	1999–2012	**−0.85 (−1.07, −0.64)**	2012–2020	**−2.39 (−2.75, −2.04)**	N/A	N/A
Liver and intrahepatic bile	**1.74 (1.57, 1.92)**	1999–2013	**2.74 (2.56, 2.93)**	2013–2020	−0.23 (−0.65, 0.19)	N/A	N/A
Lung and bronchus	**−3.49 (−3.62, −3.35)**	1999–2005	**−2.02 (−2.31, −1.73)**	2005–2013	**−3.14 (−3.38, −2.90)**	2013–2020	**−5.11 (−5.35, −4.88)**
Myeloma	**−1.10 (−1.41, −0.78)**	1999–2016	**−0.85 (−1.02, −0.69)**	2016–2020	**−2.11 (−3.75, −0.45)**	N/A	N/A
Non‐Hodgkin lymphoma	**−2.58 (−2.75, −2.41)**	1999–2006	**−3.28 (−3.74, −2.82)**	2006–2020	**−2.23 (−2.38, −2.08)**	N/A	N/A
Pancreas	**0.13 (0.06, 0.20)**	1999–2020	**0.13 (0.06, 0.20)**	N/A	N/A	N/A	N/A
Prostate	**−2.48 (−2.68, −2.27)**	1999–2013	**−3.39 (−3.57, −3.21)**	2013–2020	**−0.62 (−1.19, −0.05)**	N/A	N/A
Stomach	**−2.92 (−3.05, −2.78)**	1999–2006	**−3.57 (−3.87, −3.27)**	2006–2020	**−2.59 (−2.74, −2.44)**	N/A	N/A
Urinary bladder	**−0.61 (−0.83, −0.39)**	1999–2014	−0.07 (−0.24, 0.11)	2014–2020	**−1.94 (−2.63, −1.24)**	N/A	N/A
Rural							
All malignant cancers	**−1.43 (−1.47, −1.39)**	1999–2020	**−1.43 (−1.47, −1.39)**	N/A	N/A	N/A	N/A
Colon and rectum	**−2.01 (−2.20, −1.83)**	1999–2009	**−2.66 (−2.95, −2.36)**	2009–2020	**−1.43 (−1.70, −1.15)**	N/A	N/A
Kidney and renal pelvis	**−0.33 (−0.51, −0.14)**	1999–2020	**−0.33 (−0.51, −0.14)**	N/A	N/A	N/A	N/A
Leukemia	**−1.12 (−1.29, −0.95)**	1999–2020	**−1.12 (−1.29, −0.95)**	N/A	N/A	N/A	N/A
Liver and intrahepatic bile	**2.41 (1.59, 3.25)**	1999–2007	**1.87 (0.66, 3.10)**	2007–2012	**5.05 (2.06, 8.12)**	2012–2020	**1.34 (0.49, 2.20)**
Lung and bronchus	**−2.70 (−2.98, −2.43)**	1999–2007	**−1.47 (−1.86, −1.08)**	2007–2014	**−2.56 (−3.14, −1.97)**	2014–2020	**−4.49 (−5.08, −3.90)**
Myeloma	**−0.91 (−1.14, −0.68)**	1999–2020	**−0.91 (−1.14, −0.68)**	N/A	N/A	N/A	N/A
Non‐Hodgkin lymphoma	**−2.13 (−2.30, −1.95)**	1999–2020	**−2.13 (−2.30, −1.95)**	N/A	N/A	N/A	N/A
Pancreas	**0.66 (0.52, 0.81)**	1999–2020	**0.66 (0.52, 0.81)**	N/A	N/A	N/A	N/A
Prostate	**−2.54 (−2.78, −2.30)**	1999–2014	**−3.49 (−3.68, −3.31)**	2014–2020	−0.11 (−0.90, 0.69)	N/A	N/A
Stomach	**−2.75 (−3.20, −2.30)**	1999–2007	**−3.75 (−4.68, −2.81)**	2007–2020	**−2.13 (−2.66, −1.60)**	N/A	N/A
Urinary bladder	0.08 (−0.24, 0.41)	1999–2010	**0.77 (0.26, 1.27)**	2010–2020	**−0.66 (−1.15, −0.18)**	N/A	N/A

Segments were chosen by Joinpoint regression. Boldfaced cell indicates statistical significance (*p* < 0.05).

*Abbreviations*: AAPC, average annual percentage change; APC, annual percentage change; N/A, not applicable.

**TABLE 3:** Annual percentage changes in cancer death rates by rural–urban status and cancer site among **females** aged ≥25 years in the United States, 1999–2020.

Cause of Death	AAPC	Segment 1		Segment 2		Segment 3	
Urban							
All malignant cancers	**−1.56 (−1.64, −1.48)**	1999–2003	**−1.24 (−1.55, −0.93)**	2003–2016	**−1.52 (−1.58, −1.47)**	2016–2020	**−2.29 (−2.29, −1.70)**
Female breast	**−1.69 (−1.81, −1.56)**	1999–2013	**−1.93 (−2.03, −1.83)**	2013–2020	**−1.21 (−1.56, −0.86)**	N/A	N/A
Cervix uteri	**−1.28 (−1.51, −1.06)**	1999–2004	**−2.66 (−3.50, −1.81)**	2004–2020	**−0.85 (−1.02, −0.68)**	N/A	N/A
Colon and rectum	**−2.74 (−2.91, −2.57)**	1999–2012	**−3.13 (−3.32, −2.95)**	2012–2020	**−2.09 (−2.46, −1.72)**	N/A	N/A
Corpus and uterus, NOS	**1.11 (0.83, 1.40)**	1999–2008	0.23 (−0.13, 0.60)	2008–2016	**2.20 (1.70, 2.70)**	2016–2020	0.95 (−0.08, 1.98)
Kidney and renal Pelvis	**−1.38 (−1.83, −0.93)**	1999–2003	0.56 (−1.67, 2.84)	2003–2020	**−1.83 (−2.12, −1.55)**	N/A	N/A
Leukemia	**−1.58 (−1.90, −1.26)**	1999–2004	**−1.75 (−2.58, −0.92)**	2004–2011	**−0.76 (−1.44, −0.07)**	2011–2020	**−2.12 (−2.53, −1.70)**
Liver and intrahepatic Bile	**1.60 (1.27, 1.93)**	1999–2008	**1.54 (1.11, 1.98)**	2008–2014	**2.93 (2.03, 3.85)**	2014–2020	0.37 (−0.23, 0.98)
Lung and bronchus	**−2.25 (−2.42, −2.08)**	1999–2006	**−0.37 (−0.70, −0.04)**	2006–2014	**−2.16 (−2.45, −1.88)**	2014–2020	**−4.51 (−4.89, −4.13)**
Myeloma	**−1.57 (−2.10, −1.04)**	1999–2009	**−2.11 (−2.53, −1.70)**	2009–2014	0.39 (−1.50, 2.33)	2014–2020	**−2.30 (−3.37, −1.22)**
Non‐Hodgkin lymphoma	**−3.19 (−3.38, −3.01)**	1999–2004	**−3.97 (−4.61, −3.32)**	2004–2020	**−2.95 (−3.11, −2.79)**	N/A	N/A
Ovary	**−1.96 (−2.25, −1.66)**	1999–2003	0.25 (−0.66, 1.16)	2003–2016	**−2.25 (−2.44, −2.05)**	2016–2020	**−3.17 (−4.43, −1.90)**
Pancreas	0.11 (−0.01, 0.24)	1999–2008	**0.37 (0.13, 0.60)**	2008–2020	−0.08 (−0.23, 0.07)	N/A	N/A
Stomach	**−2.37 (−2.58, −2.17)**	1999–2008	**−3.05 (−3.38, −2.71)**	2008–2020	**−1.87 (−2.15, −1.58)**	N/A	N/A
Urinary bladder	**−0.81 (−1.02, −0.61)**	1999–2020	**−0.81 (−1.02, −0.61)**	N/A	N/A	N/A	N/A
Rural							
All malignant cancers	**−0.93 (−1.03, −0.82)**	1999–2015	**−0.80 (−0.88, −0.73)**	2015–2020	**−1.31 (−1.73, −0.90)**	N/A	N/A
Female breast	**−1.32 (−1.49, −1.14)**	1999–2008	**−1.68 (−2.00, −1.36)**	2008–2020	**−1.04 (−1.26, −0.82)**	N/A	N/A
Cervix uteri	**−1.15 (−1.80, −0.51)**	1999–2003	**−4.80 (−7.83, −1.66)**	2003–2020	−0.28 (−0.69, 0.13)	N/A	N/A
Colon and rectum	**−2.10 (−2.40, −1.80)**	1999–2005	**−2.98 (−3.94, −2.02)**	2005–2020	**−1.74 (−1.97, −1.51)**	N/A	N/A
Corpus and uterus, NOS	**0.92 (0.67, 1.17)**	1999–2020	**0.92 (0.67, 1.17)**	N/A	N/A	N/A	N/A
Kidney and renal pelvis	**−0.69 (−0.99, −0.38)**	1999–2020	**−0.69 (−0.99, −0.38)**	N/A	N/A	N/A	N/A
Leukemia	**−1.35 (−1.54, −1.17)**	1999–2020	**−1.35 (−1.54, −1.17)**	N/A	N/A	N/A	N/A
Liver and intrahepatic bile	**1.73 (0.62, 2.85)**	1999–2008	0.23 (−1.03, 1.50)	2008–2013	**4.80 (0.68, 9.09)**	2013–2020	**1.52 (0.03, 3.02)**
Lung and bronchus	**−0.79 (−1.10, −0.48)**	1999–2007	**1.03 (0.56, 1.50)**	2007–2014	**−1.10 (−1.75, −0.44)**	2014–2020	**−2.80 (−3.45, −2.14)**
Myeloma	**−1.40 (−1.67, −1.13)**	1999–2020	**−1.40 (−1.67, −1.13)**	N/A	N/A	N/A	N/A
Non‐Hodgkin lymphoma	**−2.98 (−3.24, −2.73)**	1999–2004	**−4.19 (−5.11, −3.27)**	2004–2020	**−2.60 (−2.83, −2.38)**	N/A	N/A
Ovary	**−1.78 (−2.28, −1.28)**	1999–2004	0.47 (−1.13, 2.10)	2004–2013	**−1.92 (−2.59, −1.25)**	2013–2020	**−3.19 (−4.01, −2.36)**
Pancreas	**0.55 (0.45, 0.65)**	1999–2020	**0.55 (0.45, 0.65)**	N/A	N/A	N/A	N/A
Stomach	**−2.16 (−2.50, −1.83)**	1999–2020	**−2.16 (−2.50, −1.83)**	N/A	N/A	N/A	N/A
Urinary bladder	−0.04 (−0.33, 0.25)	1999–2020	−0.04 (−0.33, 0.25)	N/A	N/A	N/A	N/A

*Note*: Segments were chosen by joinpoint regression. Boldfaced cell indicates statistical significance (p < 0.05).

*Abbreviations*: AAPC, average annual percentage change; APC, annual percentage change; N/A, not applicable.

**FIGURE 1:**
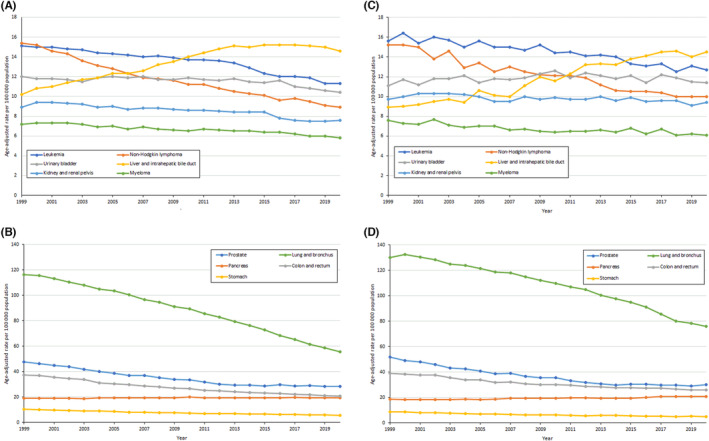
Trends in age‐adjusted cancer deaths rates among males by rural–urban (urban, A and B; rural, C and D) cancer site from 1999 to 2020.

**FIGURE 2:**
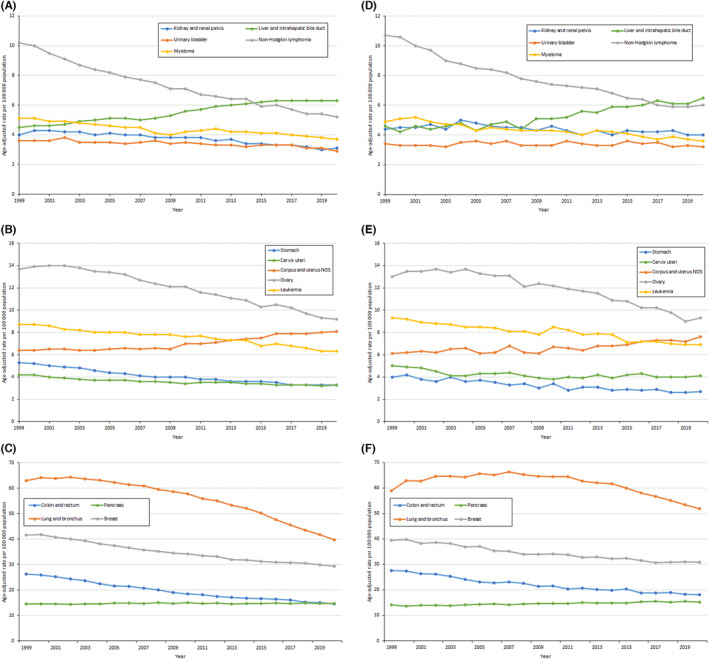
Trends in age‐adjusted cancer deaths rates among females by rural–urban (urban, A, B, and C; rural, D, E, and F) cancer site from 1999 to 2020.

For cancer‐specific trends, there were reductions in death rates for most cancer sites among males except for pancreas and liver cancers (Figure 1). The rate of increase in deaths from liver and pancreas cancers was more remarkable for males in rural communities than males in urban areas (Table 2). Furthermore, while an inflection point was observed for liver cancer death rates such that rates were stable among males in urban areas between 2013 and 2020 (APC, −0.23%; 95% CI −0.65, 0.19), liver cancer mortality rates continued to rise between 2012 and 2020 among males in rural areas (APC, 1.34; 95% CI, 0.49, 2.20). Deaths from pancreas cancer increased faster among rural males (0.66%/year) than urban males (0.13%/year). For cancer sites with decreasing rates, the decline rates were greater for males in urban compared to rural areas. Similarly, among females, there were decreases in rates for most cancer sites except the liver, uterus, and pancreas (Figure 2). Unlike males, the overall rate of increase (AAPC) in death rates for liver and uterus cancers was not higher among females from rural compared to urban areas. Nonetheless, death rates for liver, uterus, and pancreas cancers continue to increase in rural females but not urban females (Table 3).

### 3.3 Racial/Ethnic disparities in cancer mortality trends

Overall, cancer death rates decreased in all racial and ethnic populations; however, the rural–urban differences varied by race/ethnicity (Table 4). Results from the parallelism test show that NHW and NHB males and females' rates of decline were greater and secular trends were different for urban areas compared to rural areas (Table S2). Conversely, the rate of decline was more remarkable for Hispanics in rural areas compared to Hispanics in urban areas. The same associations were seen for API males but not females (*p*‐value parallelism test = 0.5780). For AI/AN males and females, decline rates and trends were not different between urban and rural populations.

**TABLE 4:** Annual percentage changes in cancer death rates by rural–urban, sex and race/ethnicity among persons aged ≥25 years in the United States, 1999–2020.

	Average APC from 1999 to 2020	Segment 1		Segment 2		Segment 3	
*Urban*							
Males							
NHW	**−1.78 (−1.84, −1.72)**	1999–2014	**−1.67 (−1.72, −1.62)**	2014–2020	**−2.06 (−2.25, −1.86)**	N/A	N/A
NHB	**−2.62 (−2.66, −2.57)**	1999–2020	**−2.62 (−2.66, −2.57)**	N/A	N/A	N/A	N/A
Hispanics	**−1.59 (−1.68, −1.50)**	1999–2020	**−1.59 (−1.68, −1.50)**	N/A	N/A	N/A	N/A
API	**−1.65 (−1.82, −1.47)**	1999–2010	**−1.31 (−1.58, −1.03)**	2010–2020	**−2.02 (−2.26, −1.77)**	N/A	N/A
AI/AN	**−0.78 (−1.31, −0.24)**	1999–2010	0.76 (−0.10, 1.63)	2010–2020	**−2.44 (−3.18, −1.70)**	N/A	N/A
Females							
NHW	**−1.45 (−1.53, −1.37)**	1999–2003	**−1.01 (−1.31, −0.71)**	2003–2016	**−1.42 (−1.48, −1.37)**	2016–2020	**−1.96 (−2.28, −1.64)**
NHB	**−1.62 (−1.74, −1.50)**	1999–2012	**−1.49 (−1.62, −1.36)**	2012–2020	**−1.83 (−2.09, −1.58)**	N/A	N/A
Hispanics	**−0.94 (−1.01, −0.88)**	1999–2020	**−0.94 (−1.01, −0.88)**	N/A	N/A	N/A	N/A
API	**−1.09 (−1.22, −0.95)**	1999–2020	**−1.09 (−1.22, −0.95)**	N/A	N/A	N/A	N/A
AI/AN	**−1.02 (−1.63, −0.40)**	1999–2009	0.05 (−1.04, 1.15)	2009–2020	**−1.97 (−2.75, −1.19)**	N/A	N/A
*Rural*							
Males							
NHW	**−1.29 (−1.33, −1.24)**	1999–2020	**−1.29 (−1.33, −1.24)**	N/A	N/A	N/A	N/A
NHB	**−2.29 (−2.40, −2.17)**	1999–2020	**−2.29 (−2.40, −2.17)**	N/A	N/A	N/A	N/A
Hispanics	**−2.20 (−2.53, −1.87)**	1999–2020	**−2.20 (−2.53, −1.87)**	N/A	N/A	N/A	N/A
API	**−2.80 (−3.48, −2.13)**	1999–2020	**−2.80 (−3.48, −2.13)**	N/A	N/A	N/A	N/A
AI/AN	**−1.39 (−1.78, −0.99)**	1999–2020	**−1.39 (−1.78, −0.99)**	N/A	N/A	N/A	N/A
Females							
NHW	**−0.83 (−0.95, −0.70)**	1999–2015	**−0.71 (−0.80, −0.63)**	2015–2020	**−1.18 (−1.66, −0.70)**	N/A	N/A
NHB	**−1.23 (−1.36, −1.10)**	1999–2020	**−1.23 (−1.36, −1.10)**	N/A	N/A	N/A	N/A
Hispanics	**−1.32 (−1.49, −1.14)**	1999–2020	**−1.32 (−1.49, −1.14)**	N/A	N/A	N/A	N/A
API	**−1.31 (−1.76, −0.86)**	1999–2020	**−1.31 (−1.76, −0.86)**	N/A	N/A	N/A	N/A
AI/AN	**−0.62 (−1.16, −0.08)**	1999–2006	1.17 (−0.34, 2.70)	2006–2020	**−1.51 (−1.95, −1.06)**	N/A	N/A

*Note*: Segments were chosen by joinpoint regression. Boldfaced cell indicates statistical significance.

Abbreviations: AI/AN, American Indian/Alaska Native; APC, annual percentage change; API, Asian and Pacific Islander; N/A, not applicable; NHB, non‐Hispanic Black; NHW, non‐Hispanic white.

## 4 DISCUSSION

Using nationwide NCHS data, this study demonstrated temporal trends and variations in cancer mortality rates across rural and urban communities in the United States. The burden of cancer deaths remained significantly higher among rural versus urban communities. Additionally, there were significant decreases in cancer death rates in the U.S. between 1999 and 2020. Decreases were observed for males and females in urban and rural areas; however, the rate of decline was slower in rural communities. While deaths from most cancers decreased during the study period, mortality rates from some obesity‐related cancers (i.e., liver, pancreas, and uterine cancers) increased, particularly among rural populations.

Our findings support prior studies showing the highest cancer mortality rates in rural areas, disparities in trends, and the magnitude of declining rates.^4^, ^18^ The observed decreases in cancer mortality rates over the study period may be attributable to effective interventions across the cancer care continuum, including smoking cessation, advancements in other cancer prevention strategies, diagnosis, and treatments. Previous studies have documented disparities in health outcomes among rural populations partially due to structural, and environmental disadvantages.^7^, ^19^, ^20^, ^21^ Rural–urban‐associated differences have been reported in terms of healthcare access, missed appointments, quality, and timeliness of cancer screening, clinical trial enrollment, and treatment facility capacity.^22^ For example, some rural communities do not have access to specialist providers and have to travel greater distances to access treatment or are likely to receive cancer treatment in smaller facilities, thus experiencing poorer outcomes.^23^ These continued disparities in the receipt of guideline‐concordant care continue to hamper efforts to reduce the urban–rural gap in cancer outcomes.

While deaths from some obesity‐related cancers (e.g., breast and colorectal) are declining, other obesity‐related cancers, including liver, pancreas, and uterine cancers are rising. The association between obesity and cancer risk and mortality is well established.^24^ Studies have suggested that the prevalence of obesity^7^ and incidence of obesity‐related cancers^8^, ^9^ is increasing in the U.S. Despite increasing risks, declining rates of some obesity‐related cancers may be a result of established routine screening schedules, for example, breast and colorectal cancers.^8^, ^10^ Nonetheless, mortality from these cancer types decreased at different rates in rural versus urban areas, probably due to slower dissemination of cancer screening and advanced cancer care in rural communities.^1^

Although mortality rates for most cancers are declining, our data showing slower decreases among rural populations compared with urban groups and rising rates of some obesity‐related cancers are concerning. These issues are exacerbated for racial and ethnic minorities. Numerous barriers have been identified, including inequitable screening access, disparities in treatment modalities, supportive services, and limited patient resources, especially in rural areas.^14^ Our results indicating larger declines in cancer death rates among NHWs and NHBs are consistent with prior studies.^11^ However, our findings suggesting that some rural racial and ethnic minorities, particularly Hispanics, may be faring better than their urban counterparts in terms of reducing cancer mortality rates are yet to be reported. The intersection of race/ethnicity and rurality is complex and important in understanding and addressing cancer disparities.^25^ There remains much to understand of the underlying causes about these disparities for them to be prevented.

A strength of this study is that we used data from a national cancer database covering the entire continental U.S. and spanning over 21 consecutive years. Nevertheless, our study has limitations, including the potential for misclassification of cause of death, and race/ethnicity on death certificates. In addition, broad groupings of race and ethnicity can mask variations within groups. Furthermore, we likely underestimated the rates among AI/AN, especially for more rural areas. However, our analyses comparing trends (urban vs. rural) within racial/ethnic sub‐groups are internally validated. Moreover, the impact of the COVID‐19 pandemic on cancer death rates is still evolving. The observed decreases in cancer death rates in the current study may slow in future years due to the COVID‐19 pandemic, which has disproportionately affected Black communities in the United States. Our study period does not extend through to the pandemic. Future studies are required to assess the effect of COVID‐19 on cancer mortality rates overall and on rural versus urban communities.

In conclusion, despite overall declines in cancer mortality, rural populations continue to have higher cancer mortality rates than urban areas, particularly minority populations residing in rural areas. Increasing death rates from liver and pancreas cancers (and uterine cancers in females) are also likely to be aggravated further by the COVID‐19 pandemic and hospital closures in rural areas. Eliminating these disparities in cancer mortality will require equitable access to cancer preventive services, including vaccinations, early diagnosis, and timely guideline‐adherent cancer care.

## AUTHOR CONTRIBUTIONS

**Itunu Sokale:** Investigation (equal); writing – original draft (lead); writing – review and editing (lead). **Syed Ahsan Raza:** Investigation (equal); writing – review and editing (equal). **Aaron Peter Thrift:** Conceptualization (lead); data curation (lead); formal analysis (lead); funding acquisition (lead); investigation (equal); methodology (equal); supervision (lead); visualization (lead); writing – original draft (supporting); writing – review and editing (lead).

## FUNDING INFORMATION

This work was funded (in part) by Research Training Awards from the Cancer Prevention & Research Institute of Texas (CPRIT) for the Systems Epidemiology of Cancer Training (SECT) Program (RP210037; PI: A. Thrift).

## CONFLICT OF INTEREST STATEMENT

The authors declare no potential conflicts of interest.

## ETHICS STATEMENT

The analysis was exempted from ethical review since National Center for Health Statistics was open‐access and de‐identifiable (The detailed information of data could be found elsewhere: https://www.cdc.gov/nchs/index.htm).

## Supporting information


Table S1.

## Data Availability

Data are available from the corresponding author upon tenable request and with permission of the National Center for Health Statistics database.
