# Supplementary material for: Disparities in cancer mortality patterns: A comprehensive examination of U.S. rural and urban adults, 1999–2020
Source: Cancer Med. 2023 Aug 10;12(18):18988–98. doi: 10.1002/cam4.6451 (PMC10557857; doi:10.1002/cam4.6451)
Supplement: Supplementary file 1 — Table S1. [file CAM4-12-18988-s001.docx]

**Supplementary Table 1.** Cancer site and associated ICD-10 codes

| **SEER Cause of Death Label** | **ICD-10** |
| --- | --- |
| All Malignant Cancers | C00-C97 |
| Breast | C50 |
| Cervix Uteri | C53 |
| Colon and Rectum | C18, C20 |
| Corpus and Uterus, NOS | C54-C55 |
| Kidney and Renal Pelvis | C64-C65 |
| Leukemia | C91-C95 |
| Liver and Intrahepatic Bile | C22 |
| Lung and Bronchus | C34 |
| Myeloma | C90 |
| Non-Hodgkin Lymphoma | C82, C83, C85 |
| Ovary | C56 |
| Pancreas | C25 |
| Prostate | C61 |
| Stomach | C16 |
| Urinary Bladder | C67 |

*Abbreviations:* ICD-10, International Statistical Classification of Diseases and Related Health Problems, Tenth Revision; NOS, not otherwise specified; SEER, Surveillance, Epidemiology, and End Results.

**Supplementary Table 2.** Comparisons of average annual percentage changes in cancer death rates between racial/ethnic groups according to rural-urban status

|  |  | P-value parallelism test | AAPC Comparison* |
| --- | --- | --- | --- |
| Males |  |  |  |
|  | NHW | 0.0002 | -0.49 (-0.57, -0.42) |
|  | NHB | 0.0002 | -0.33 (-0.45, -0.21) |
|  | Hispanics | 0.0067 | 0.61 (0.29, 0.93) |
|  | API | 0.0324 | 1.16 (0.50, 1.81) |
|  | AI/AN | 0.0160 | 0.61 (-0.04, 1.26) |
| Females |  |  |  |
|  | NHW | 0.0002 | -0.62 (-0.77, -0.48) |
|  | NHB | 0.0002 | -0.39 (-0.56, -0.22) |
|  | Hispanics | 0.0111 | 0.37 (0.20, 0.55) |
|  | API | 0.5780 | - (parallel) |
|  | AI/AN | 0.6604 | - (parallel) |

*A negative value indicates a higher rate of decrease in urban areas vs. rural areas.
